# Characterization of Magnitude and Antigen Specificity of HLA-DP, DQ, and DRB3/4/5 Restricted DENV-Specific CD4+ T Cell Responses

**DOI:** 10.3389/fimmu.2019.01568

**Published:** 2019-07-05

**Authors:** Alba Grifoni, Eugene Moore, Hannah Voic, John Sidney, Elizabeth Phillips, Ramesh Jadi, Simon Mallal, Aruna D. De Silva, Aravinda M. De Silva, Bjoern Peters, Daniela Weiskopf, Alessandro Sette

**Affiliations:** ^1^Division of Vaccine Discovery, La Jolla Institute for Immunology, La Jolla, CA, United States; ^2^Institute for Immunology and Infectious Diseases, Murdoch University, Perth, WA, Australia; ^3^Department of Microbiology and Immunology, University of North Carolina School of Medicine, Chapel Hill, NC, United States; ^4^Genetech Research Institute, Colombo, Sri Lanka; ^5^Department of Medicine, University of California, San Diego, San Diego, CA, United States

**Keywords:** DENV, CD4+T cells, HLA-DP, HLA-DQ, HLA-DRB3/4/5, adaptive immunity

## Abstract

**Background:** Dengue Virus (DENV) associated disease is a major public health problem. Assessment of HLA class II restricted DENV-specific responses is relevant for immunopathology and definition of correlates of protection. While previous studies characterized responses restricted by the HLA-DRB1 locus, the responses associated with other class II loci have not been characterized to date. Accordingly, we mapped HLA-DP, DQ, and DRB3/4/5 restricted DENV-specific CD4 T cell epitopes in PBMCs derived from the DENV endemic region Sri Lanka.

**Methods:** We studied 12 DP, DQ, and DRB3/4/5 alleles that are commonly expressed and provide worldwide coverage >82% for each of the loci analyzed and >99% when combined. CD4+ T cells purified by negative selection were stimulated with pools of HLA-predicted binders for 2 weeks with autologous APC. Epitope reactive T cells were enumerated using IFNγ ELISPOT assay. This strategy was previously applied to identify DRB1 restricted epitopes. In parallel, membrane expression levels of HLA-DR, DP, and DQ proteins was assessed using flow cytometry.

**Results:** Epitopes were identified for all DP, DQ, and DRB3/4/5 allelic variants albeit with magnitudes significantly lower than the ones previously observed for the DRB1 locus. This was in line with lower membrane expression of HLA-DP and DQ molecules on the PBMCs tested, as compared to HLA-DR. Significant differences between loci were observed in antigen immunodominance. Capsid responses were dominant for DRB1/3/4/5 and DP alleles but negligible for the DQ alleles. NS3 responses were dominant in the case of DRB1/3/4/5 and DQ but absent in the case of DP. NS1 responses were prominent in the case of the DP alleles, but negligible in the case of DR and DQ. In terms of epitope specificity, repertoire was largely overlapping between DRB1 and DRB3/4/5, while DP and DQ loci recognized largely distinct epitope sets.

**Conclusion:** The HLA-DP, DQ, and DRB3/4/5 loci mediate DENV-CD4 specific immune responses of lower magnitude as compared to HLA-DRB1, consistent with their lower levels of expression. The responses are associated with distinct and characteristic patterns of immunodominance, and variable epitope overlap across loci.

## Introduction

The burden of DENV disease has dramatically increased worldwide in the past decades. Recent epidemiological data estimate that almost 400 million DENV infections occurs per year, of which 25% are symptomatic and associated with clinical presentations of various severity (1). These numbers clearly highlight the health threat that DENV represents worldwide (2, 3).

DENV-specific immune responses have been extensively studied in past years, highlighting key roles of both B and T cell responses during infection. Whether T cells play a greater role in disease protection or pathogenesis has been debated. Several recent studies have shown that both CD4 and CD8 T cells can exert a protective effect in the context of DENV infections (4–10). This is in contrast to an earlier hypothesis that suboptimal cross-reactive memory T cells may impair viral control upon secondary heterologous infections (11–13).

To enable comprehensive worldwide assessment of the magnitude, antigen specificity, and epitope repertoire associated with DENV-specific T cells, several studies have been carried out in the general populations of endemic areas and following vaccination. At the level of CD8 T cell responses, responses restricted by more than 30 different HLA–A and B allelic variants have been defined (6, 14–18). Several studies defined HLA class II-restricted responses both in the endemic areas of Sri Lanka and Nicaragua, and following vaccination (7, 8, 15, 17, 19–23).

HLA class II molecules are heterodimers composed of an alpha and beta chain. DRB1 is a highly polymorphic locus which encodes a beta chain which pairs with an essentially monomorphic alpha chain. The same alpha chain also pairs with the beta chains produced by the DRB3/4/5 locus. Similarly, additional alpha and beta chains are encoded by the DP and DQ loci (24, 25). Thus, in a heterozygote individual, a total of eight different HLA class II heterodimers can be expressed (two DRB1/DRA, two DRB3/4/5/DRA, two DP, and two DQs).

Our previous study of class II restricted DENV epitopes focused on responses associated with HLA DRB1 allelic variants since DRB1 is the most commonly studied human class II molecule, and DRB1 restricts the majority of defined epitopes in the literature. However, this initial focus was by definition incomplete, since it did not address the contribution of the DRB3/4/5, DP, and DQ loci. A general bias toward DRB1-restriction may be observed throughout the scientific literature. For instance, querying the Immune Epitope Database (IEDB) (https://www.iedb.org) that collects a list of experimental data on B and T cell epitopes, reveals that 1,027, 0, 8, and 0 instances of DENV epitopes restricted by HLA-DRB1, -DRB3/4/5, -DP, and -DQ loci, respectively. Thus, data available regarding DENV-epitopes restricted for DRB3/4/5, DP, and DQ loci are scarce and this represents an important gap of knowledge. In this study, we sought to characterize the contribution of DRB3/4/5, DP, and DQ and loci in DENV-specific CD4 T cell immune responses and compare it to the previously defined DRB1 locus.

## Materials and Methods

### Human Blood Samples

A total of 120 peripheral blood samples were obtained from healthy adult blood donors from the National Blood Center, Ministry of Health, Colombo, Sri Lanka in an anonymous fashion as previously reported (14). Both sexes were represented and donors ranged from 18 to 60 years of age. All protocols described herein, were approved by the institutional review boards of both LJI and Medical Faculty, University of Colombo (serving as NIH approved IRB for Genetech). Blood collection and processing was performed, as previously described (14). The blood samples were obtained before DENV serology screening. Serology screening (described in depth in the section below) identified 96 samples collected and used for this study with broad neutralization profile, suggesting that the donors had experienced one or more DENV infections prior to blood donation. Donor serology informations are shown in Supplementary Table S1.

### Serology

Serum neutralization assays were performed in Vero cells in all the donors of this study as previously reported (26). Briefly, Vero-81 cells (2 × 10^4^ cells/well, ATCC no. CCL-81) were seeded on 96-well flat-bottom tissue culture-treated plates (Greiner Sigma Aldrich), and incubated at 37°C overnight. Equal volumes of DENV viruses (DENV-1 West Pac 74, DENV-2 S-16803, DENV-3 CH54389, and DENV-4 TVP-360) and eight 4-fold serial dilutions of heat inactivated human sera were mixed and incubated for 1 h at 37°C and then transferred to 96 well-plate with complete cell monolayer. Plates were incubated for 1 h at 37°C with 5% CO_2_ for virus adsorption. After washing, an overlay medium (1%carboxymethylcellulose) (200 μl/well) was added in the wells and incubated for 48 h. Carboxymethyl cellulose solution was discarded, plates were washed and cells were fixed with 4% para formaldehyde. Plates were blocked primary antibody was added (crude mAb 4G2 pan-DENV anti-E protein and 2H_2_ PrM binding antibody in 1:300 dilutions) followed by wash and incubation with goat anti mouse IgG –HPR conjugated (1:1,000 in blocking buffer). After washing, True blue substrate was added (True Blue HRP substrate, VWR). The number of foci were visualized and quantified by automatic counting using a CTL ImmunoSpot (Cellular Technology Limited). The log_10_ of the reciprocal serum dilution was plotted against relative infection, calculated as [(# spots sample–# spots non-infected control)/(# spots (virus + normal human serum)–# spots non-infected control)], and fitted with a sigmoidal dose-response curve using GraphPad Prism software version 8.0 (La Jolla, CA). The titer of antibody (serum dilution) that achieved a 50% reduction in infection (50% neutralization titer, NT50), is expressed as the reciprocal of the serum dilution. Maximum infection was calculated from monolayers infected in the presence of normal human serum. Stringent QC rules, including the absolute sum of squares <0.2 and the coefficient of determination (R2) of the non-linear regression >0.9, were required to ensure the reliability of results. Serology results are shown in Supplementary Table S1.

### HLA Typing and Phenotype Frequency Calculations

HLA typing was performed by an ASHI-accredited laboratory at Murdoch University (Western Australia) for Class I (HLA A; B; C) and Class II (DRB1, DRB3/4/5, DQA1/DQB1, DPB1) as previously described (15, 22, 23). Allele and phenotype frequencies for individual DP/DQ alleles in Sri Lanka population have been previously described (27). Worldwide phenotype frequency were calculated as previously described and based on data available at DbMHC and allelefrequencies.net (15, 28–32). Population combined coverage for DP, DQ, and DRB345 loci was calculated as follows:

$$\begin{array}{l} Population\;coverage=\text{ A}+[\left(100-\text{A}\right)*\frac{\text{DQ coverage}}{\text{100}}]\;\\ \text{Where A}=\;DRB345\;coverage\;+[\left(100-\text{DRB345 coverage}\right)\\ \;*\frac{\text{DP coverage}}{\text{100}}] \end{array}$$

### MHC Class II Binding Predictions and Peptide Selection

A set of DENV peptides predicted to bind various DRB3/4/5 (DRB3^*^0202, DRB4^*^0101, DRB5^*^0101), DP (DPA1^*^01/DPB1^*^0401, DPA1^*^0103/DPB1^*^0201, DPA1^*^0201/DPB1^*^0101, DPA1^*^0301/DPB1^*^0402), and DQ (DQA1^*^0301/DQB1^*^0302, DQA1^*^0101/DQB1^*^0501, DQA1^*^0102/DQB1^*^0602, DQA1^*^0501/DQB1^*^0201, DQA1^*^0501/DQB1^*^0301) alleles was chosen based on criteria further described in the results section. Fifteen-mer peptides from all serotypes were predicted for their binding affinity to the selected HLA class II molecules as previously described (15, 33, 34). This resulted in the synthesis of 432, 448, and 562 predicted binding peptides for HLA-DRB3/4/5, DP, and DQ and loci, respectively, (Mimotopes, Victoria, Australia) as shown in Supplementary Table S1. For screening purposes, pools of peptides predicted to bind each of the HLA class II allelic variants were used for stimulation and deconvoluted to the single peptide level as previously reported (8, 15).

### *In vitro* Expansion of Denv-Specific T Cells and IFNγ Elispot Assay

CD4+T cells stimulation was performed as previously described (8, 15). Briefly, frozen Peripheral Blood Mononuclear Cell (PBMCs) were thawed and CD4^+^ T cells were isolated by magnetic bead negative selection and co-cultured with autologous APC derived from the positive selection in a 2:1 ratio. Cells were stimulated with DENV-specific pools kept at 37°C in 5% CO_2_, IL-2 (10 U/mL; eBioscience) was added at 4, 7, and 11 days after initial antigenic stimulation and harvested on day 14. Harvested cells were counted and plated 5 × 10^4^ CD4^+^ T cells in triplicates in the presence of the HLA-matched peptide pools used for stimulation [1 μg/ml] and the individual peptide contained in the pool used for stimulation [10 μg/ml]. When the number of cells were not sufficient to test all the peptides contained in the pool used for stimulation a factorial approach has been followed, alternatively, all the peptides were directly deconvoluted after 14 days restimulation. On day 14, no additional APC were added to the culture. After 20 h of incubation at 37°C, cells were incubated with biotinylated IFNγ mAb (mAb 7-B6-1 Mabtech, Stockholm, Sweden) for 2 h and developed as previously described (7, 14).

### Flow Cytometry

PBMCs from 10 randomly selected Sri Lankan donors were evaluated for Class II MHC expression by flow cytometry using anti-DR, -DP, or -DQ antibodies (LB3.1, B7/21, and SPV-L3 clones, respectively). Briefly, thawed PBMCs were counted and the cell suspension adjusted to 1 × 10^6^ cells/ml. Cell suspensions were washed by centrifugation in ice-cold FACS Buffer (PBS, 10% FBS) followed by incubation with unconjugated primary antibodies for 30 min at 4°C. After subsequent wash steps, cells were incubated with FITC-conjugated goat anti-mouse IgG (Jackson ImmunoResearch, Inc. West Grove, PA) for 30 min at 4°C. Additionally, control cells were incubated with isotype antibody only. Data were acquired on a BD FACSCanto™ II analyzer and Median Fluorescence Intensities (MFI) were determined using FlowJo v10 (FlowJo, LLC).

### Cluster Analysis

A List of epitopes positive in more than one donor and restricted to different HLA class II loci (DP, DQ, DRB3/4/5, and DRB1 for reference) has been clustered using identity threshold of 70% and the recommended Cluster-break method implemented in cluster tool 2.0 (35) available in IEDB (http://tools.iedb.org/cluster2/). The resultant clusters and singlets overlap across the different HLA class II loci were then graphically shown in a Venn diagram format using Venny 2.1 (http://bioinfogp.cnb.csic.es/tools/venny/index.html).

## Results

### Selection of a Set of HLA, DP, DQ, and DRB3/4/5 Alleles Enabling High Population Phenotypic Coverage

**Figure 1:**
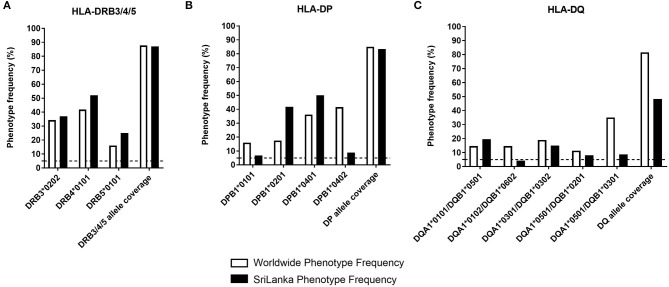
HLA class II phenotype frequencies in DP, DQ, and DRB3/4/5 loci. The HLA-DRB3/4/5 **(A)**, -DP **(B)**, and -DQ **(C)** phenotypic frequency and allele coverage worldwide (white bars), is compared to the one observed in Sri Lanka donors (*n* = 714, black bars) and based on previously published data (27).

To assess the contribution of DRB3/4/5, DP, and DQ loci in restricting DENV-specific responses, we selected three to five of the most frequent alleles worldwide for each locus (Figure 1, white bars). In the case of the DRB3/4/5, these allelic variants were DRB3^*^0202, DRB4^*^0101, and DRB5^*^0101. These alleles are found at frequencies in the 25–52% range, and combined afford coverage of 87% (Figure 1A, white bars). In the case of DP, we selected DPB1^*^0401, DPB1^*^0201, DPB1^*^0101, and DPB1^*^0402. While the alpha chain of DP molecule is also variable, haplotype, and typing data is not generally available, the alpha chain is not very polymorphic, and the polymorphisms are generally conservative. These alleles are found at frequencies in the 16–41.6% range, and combined afford a coverage of 85% (Figure 1B, white bars). In the case of DQ we selected DQA1^*^0301/DQB1^*^0302, DQA1^*^0101/DQB1^*^0501, DQA1^*^0102/DQB1^*^0602, DQA1^*^0501/DQB1^*^0201, and DQA1^*^0501/DQB1^*^0301. These alleles are found at frequencies in the 11.3–35.1% range, and combined afford coverage of 81.6% (Figure 1C, white bars). The phenotypic coverage provided when the three loci are combined (assuming no linkage disequilibrium as a first approximation) was projected at >99%.

In our studies, we utilized PBMC from the general population of Colombo, Sri Lanka. Accordingly we compared worldwide frequencies with the ones observed in the Sri Lankan population (Figure 1, black bars) based on HLA typing studies we previously reported (27). In general, the selected alleles afforded good population coverage also in the Sri Lankan population in the 48.2–83.4% range across loci. The phenotypic coverage provided when the three loci are combined was projected at >98%.

For each allele, we then selected five to seven PBMC samples for an immunogenicity screen as described in the following section.

### Identification of DENV-Derived Putative DRB3/4/5, DP, and DQ Restricted Epitopes

We synthesized and screened sets of epitopes candidates restricted for each of the selected HLA alleles derived from each of the main four DENV serotypes, based on their predicted capacity to bind each HLA allelic variant, as previously described (8, 15). The number of predicted epitopes for each allele combinations studied in Sri Lanka are shown in Supplementary Table S2. Each of the peptide sets was screened in 5–7 HLA matched donors, serologically screened, and confirmed to be exposed to one or more DENV serotype prior to blood drawn (Supplementary Table S1). A complete list of all epitopes identified in this study, including HLA restriction, response frequency, and DENV protein composition has been submitted to the IEDB (http://www.iedb.org/subid/1000789) and is available for reference in Supplementary Table S3.

We identified 111, 123, and 106 epitopes restricted by HLA-DRB3/4/5, -DP, and –DQ, respectively. From the 340 epitopes identified, 250 were newly identified and not previously reported in the IEDB. For each HLA class II allele belonging to DRB3/4/5, DP, or DQ loci, Table 1 shows the breadth and the magnitude of the responses normalized by the number of instances each allele- specific peptide set combination has been tested. On average, we identified a repertoire breadth of five epitopes per allele tested, ranging from 0 to 34 epitopes recognized in a single donor and with an average response per epitope of 891 SFC/10^6^ PBMCs (Table 1). The percentage of epitopes as a function of the DENV serotype is also shown; we considered conserved epitopes when sequences shared more than 70% of homology after cluster analysis (35). We found that the majority of the epitopes recognized are conserved across the DENV serotype when 70% homology cutoff was used with an average of 60% of the epitope recognized being conserved ranging between 55 and 70% across the DRB3/4/5, DP, and DQ loci. Overall, a similar DENV serotype recognition was observed across the loci analyzed (Table 1).

**Table 1:** Characteristics of DENV-specific responses in DRB3/4/5, DP, and DQ loci.

**Locus**	**Allele**	No. of times tested^*^	**Avg no. of epitopes per HLA allele^#^**	**Avg response per epitope^#^**	**Avg response per HLA allele^#^**	**% of epitopes per HLA allele per serotype**				
						**DENV1 (%)**	**DENV2 (%)**	**DENV3 (%)**	**DENV4 (%)**	**Conserved (70% homology)**
DRB3/4/5	DRB3^*^0202	6	2	162	271	10	10	10	0	70
	DRB4^*^0101	6	5	591	2,661	11	11	4	0	74
	DRB5^*^0101	7	11	1,921	20,309	3	11	8	12	66
DP	DPB1^*^0101	6	4	622	2,694	8	4	8	8	73
	DPB1^*^0201	6	3	308	873	6	6	18	6	65
	DPB1^*^0401	5	8	892	7,166	10	0	20	10	61
	DPB1^*^0402	5	8	1,165	9,087	23	10	18	8	41
DQ	DQA1^*^0101/DQB1^*^0501	5	6	1,514	9,525	12	18	3	9	59
	DQA1^*^0102/DQB1^*^0602	7	5	1,093	5,911	13	20	5	13	50
	DQA1^*^0301/DQB1^*^0302	6	3	1,375	4,082	11	21	0	26	42
	DQA1^*^0501/DQB1^*^0201	6	1	364	395	14	29	0	0	57
	DQA1^*^0501/DQB1^*^0301	6	1	680	635	17	0	0	17	67
	**Avg**	**6**	**5**	**891**	**5,301**	**11**	**12**	**8**	**9**	**60**

* *Factorial approach has been used for this study. Responses have been normalized based on the number of instances a full HLA allele-specific peptide set has been tested*.

# *Data calculated as (SFC/10^6^ CD4)*.

In terms of magnitude of responses, we calculated the average magnitude of response per donor (or multiple donors used to test the complete set of predicted peptide per HLA allele) by summing the total responses for each of the various HLA class II alleles studied and dividing the sum by the number of tested donors, using the same methodology as previously reported for DRB1 alleles (15). As we sought to compare the DP, DQ, DRB3/4/5 data with the ones previously published on DRB1 (8, 15), we kept consistent both experimental and analysis strategies.

**Figure 2:**
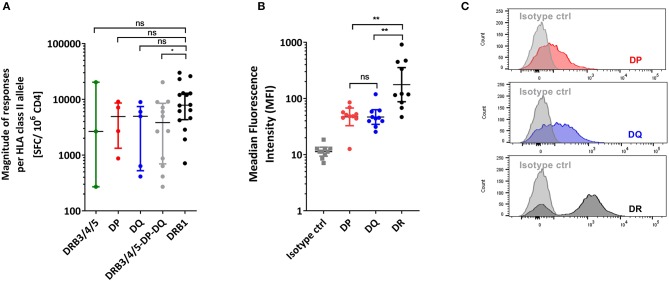
HLA class II loci comparison. **(A)** Magnitude of CD4+T cell responses per HLA class II allele expressed as SFC/10^6^ CD4 cells/donor after *in vitro* stimulation. **(B)** Membrane expression of HLA-DR, DP, DQ molecules in PBMCs expressed as Median Fluorescence Intensity (MFI). All statistical analysis were performed using unpaired non-parametric Mann-Whitney test. ^*^p < 0.05, ^**^p < 0.01, ns, not significant. **(C)** Representative overlay histograms derived from one donor PBMCs staining showing the membrane expression of isotype control (gray) overlayed with HLA-DR (black), DP (blue) or DQ (red) molecules, respectively.

Figure 2A shows the average magnitude of responses, where each allele represent a data point. Despite a rather substantial range of responses the median magnitude for responses associated with the DRB3/4/5, DP, and DQ loci were similar, with values of ~4,000 SFC/donor. In particular, the range was highest for DRB3/4/5, with the DRB3^*^02:02 allele having the highest and DRB4^*^01:01 the lowest reactivity. These results represent the first description of DENV-derived putative DRB3/4/5, DP, and DQ restricted epitopes.

### Comparison of Magnitude of HLA DRB3/4/5, DP, and DQ vs. HLA DRB1 Responses

Next we compared the responses in the case of the DRB3/4/5, DP, and DQ loci with responses previously defined for different DRB1 alleles (15), using the exact same methodology. For each allele, the sum of magnitude of responses was added and divided by the number of donors tested to calculate the average magnitude/donor response. Figure 2A shows the average magnitude of responses, for the DRB3/4/5, DP, and DQ loci, compared to data from 16 DRB1 alleles previously published (15) which is shown here for reference purposes. HLA-DRB1 was associated with higher magnitude of responses (about 2-fold) compared to the other HLA class II loci combined (Mann Whitney, *p* = 0.0473; Figure 2A).

We hypothesized that this increased magnitude of responses could be due to a difference in cells surface expression of the three different HLA class II loci. Indeed, several reports indicate that DRB1 gene products are expressed at levels 5–10 higher than those encoded by the DRB3/4/5, DP, and DQ loci (36). To verify that this was also the case in our experimental system, PBMCs derived from 10 randomly selected Sri Lanka blood bank donors, were stained for the expression of HLA-DRA (the antibody used could not therefore, discriminate between DRB1 and DRB3/4/5 loci) (37), DP, and DQ (38). As expected, we did observe significantly higher (about 5-fold) expression of DR in comparison with DP and DQ (Wilcoxon test, DP vs. DR *p* = 0.020; DQ vs. DR *p* = 0.002) (Figures 2B,C). Overall, the HLA-DRB1 locus is associated with higher magnitude of responses, which correlates with higher expression level on cell surfaces among the various HLA class II loci.

### Differences and Similarities in Immunodominance Patterns of DENV-Specific CD4+T Cells Responses Protein as a Function of HLA-Class II Loci

**Figure 3:**
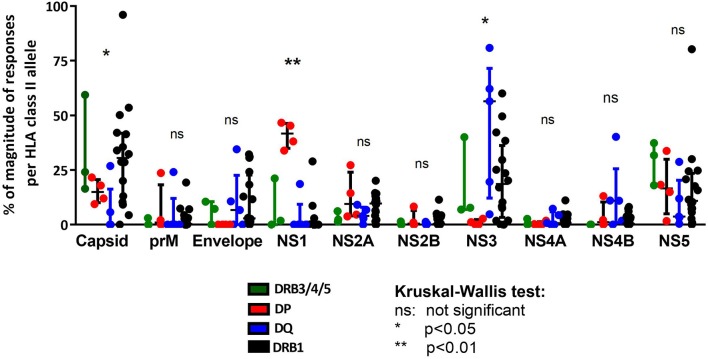
Immunodominance protein pattern of HLA class II loci comparison. Protein immunodominance observed in the previously published HLA-DRB1 locus as reference (black) (15) is compared with HLA-DP (red), HLA-DQ (blue), and HLA-DRB3/4/5 loci (green). Each dot represents the percentage of magnitude of responses per allele in the respective locus divided for DENV protein composition. Statistical analysis per protein have been performed using one-way ANOVA non-parametric Kruskal-Wallis test.

We next analyzed the pattern of immunodominance of responses as a function of the different HLA class II loci. Specifically, for each HLA allelic variant we calculated the fraction of the total response directed against each of the 10 antigens encoded by the DENV polyprotein. We next investigated whether locus-specific differences were present (Figure 3). For the sake of comparison, we also included previously reported data, related to 16 alleles of the DRB1 locus which showed a preferential response toward capsid, NS3 and NS5 proteins (Figure 3). We emphasize that the DRB1 data was previously published (15) and is shown here only for reference purposes. This analysis revealed difference in the pattern of immunodominance for some of the DENV antigens (Figure 3). Pie charts showing the fraction of the total response corresponding to each DENV protein are also shown to highlight these differences (Supplementary Figure S1). Specifically, while Capsid was dominantly recognized by DRB1 and DRB3/4/5 (accounting for about 25% of the response), it was only marginally recognized by DQ alleles (*p* = 0.0442 in a Kruskal-Wallis test). The NS1 antigen was dominantly recognized by DP alleles, but only marginally by DRB1, DRB3/4/5, and DQ loci (*p* = 0.0028 in a Kruskal-Wallis test). When looking at the NS1- specific immunodominance observed in the context of DP locus, we also identified an immunodominant NS1 region (NS1_933−949_ DYGFGVFTTNIWLKLRE) recognized across the four DP alleles (Supplementary Table S3). Conversely the NS3 antigen was not recognized by DP responses, but otherwise dominantly recognized by DRB1, DRB3/4/5, and DQ loci (*p* = 0.0279 in a Kruskal-Wallis test).

### DENV HLA Class II-Restricted Epitopes Overlap Across the Different Loci

**Figure 4:**
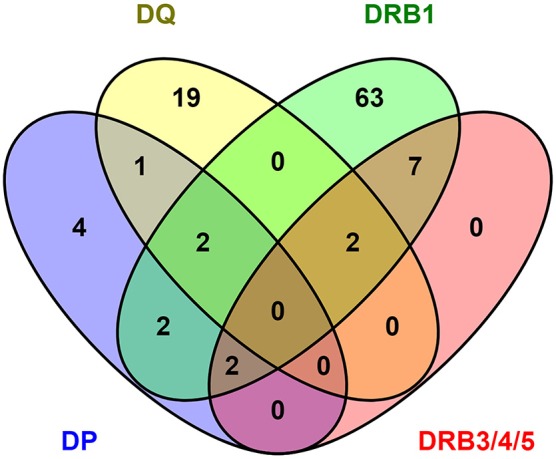
DENV epitope overlap across HLA class II loci. The number of epitopes positive in more than one donor have been clustered using cluster 2.0 (35) (http://tools.iedb.org/cluster2/) and shown as Venn diagram using Venny 2.1 (http://bioinfogp.cnb.csic.es/tools/venny/index.html).

As a final analysis of similarity and differences in the CD4 responses to DENV as a function of different HLA loci, we examined the responses for overlap at the level of the epitope repertoires associated with each locus. For this purpose, we considered epitopes that were positive in at least two of the donors tested for each allele, also including those previously identified in the context of DRB1 locus. As such we considered 22, 32, and 39 epitopes restricted to DP, DQ, and DRB3/4/5 restriction, respectively, for a total of 93 epitopes, and 183 epitopes restricted to DRB1 and previously identified in the same population (15). To account for minor sequence variations and difference in the peptide frame, these epitopes were clustered using the cluster break-method at 70% of homology using cluster 2.0 tool available in IEDB (35). The results of this analysis identified 112 epitope clusters shown as a Venn diagram in Figure 4. Of those 112 clusters, 78 were recognized by DRB1 (70%). Of those 78, 11 were shared with DRB3/4/5 (14%), 6 with DP (8%), while only 4 were shared with DQ (5%). In the case of DRB3/4/5, 11 clusters were recognized (10%); all were shared with DRB1 (100%), while only two with DP (18%), and two with DQ (18%). In the case of DP, 11 clusters were recognized (10%); of those six were shared with DRB1 (54%), while only two with DRB3/4/5 (18%), and three with DQ (27%). Finally, in the case of DQ, 24 clusters were recognized (21%); of those four were shared with DRB1 (17%), two with DRB3/4/5 (8%), and three with DP (12%).

Overall, these results suggest that there is large overlap between DRB1 and DRB3/4/5 loci, with DP showing an intermediate pattern of repertoire overlap, and DQ being associated with a mostly unique repertoire.

## Discussion

Dengue Virus-specific CD4+T cells responses are important in the control of infection and strong responses were previously associated with control of DENV infection in asymptomatic individuals (39). We previously studied in the context of DENV-specific CD4+T cell responses the role of DRB1 locus in different DENV endemic populations (8, 15). In this study, we extended our analysis to DP, DQ, and DRB3/4/5 loci using the same strategy and same DENV population previously reported for DRB1 locus.

In general, in humans most defined restrictions are associated with the DRB1 locus. By querying the IEDB (www.IEDB.org) for T cell responses in human hosts that have known MHC class II restriction, we found that 4,010 instances of epitopes restricted by DRB1 molecules, in contrast to 348, 246, and 584 instances of DRB3/4/5, DP, and DQ restrictions. It is however not clear based on this analysis alone, whether this data preponderance is to be ascribed to the fact that these responses are simply less frequently studied, or whether this reflect a lower magnitude of responses/epitope numbers associated with these loci.

Significantly weaker responses and lower number epitopes were identified for DP, DQ, and DRB3/4/5 loci combined when compared to DRB1 locus. These data are also consistent with other studies which reported lower number of epitopes found in DQ and DP loci compared to DR locus in both allergy, autoimmune diseases and viral contexts (40–42). In the allergy context, we have previously shown that the frequency of locus distribution for HLA class II antigen-specific responses was the highest for DR (61 and 49% of total SFC), followed by DP (21 and 35% of total SFC), and DQ (18 and 16% of total SFC) (42).

Given the 2 log variability in the magnitude of the CD4 response depending on the MHC II allele considered, it is not obvious that DRB3/4/5 alleles induce responses of lower magnitude compared to DRB1. However, when DRB3/4/5, DP, and DQ loci are combined together we do observe a significant lower magnitude of response respect to DRB1 locus. This is consistent with the previously reported lower expression of heterodimers encoded by DP, DQ, and DRB3/4/5 (43, 44), also confirmed in our study. Specifically, we showed that HLA-DR protein (which combines expression of both DRB1 and DRB3/4/5 loci) is significantly more highly expressed on cell surface when compared to DP and DQ loci, suggesting a higher probability that epitopes DR-restricted are recognized by CD4+T cells respect to DP and DQ. This evidence is consistent with other studies showing the same pattern of protein expression in several Antigen Presenting Cells (APC) derived from PBMCs or cell lines (43, 44).

As this study evaluated only IFNγ-specific T cell responses, it cannot be excluded that epitope recognition in the context of DP, DQ, and DRB3/4/5 could induce effector T cell responses differing from IFNγ release. However, lower general reactivity of DP and DQ compared with DR was noted as well by Kwok group using tetramer reagents (personal communication), thus arguing against this possibility.

Our results also show some qualitative differences between the immunodominance of the responses restricted by the various loci. Capsid was dominantly restricted by DR but marginally by DP and DQ. NS1 is preferentially presented by DP, and NS3 responses are restricted by all loci except DP. These differences are at least in part accounted by how frequent the peptides carrying specific motifs are found in the various proteins. Further studies should be focused on explaining why NS1 is more efficiently recognized when restricted by DP compared with other loci. In any case, these data reemphasize the fact that MHC class II allele polymorphism is a key determinant of immune responsiveness. Indeed, for this exact reason MHC class II genes were originally labeled in the 1970s as “IR” or “Immune Response” genes (45).

Finally, we considered the epitope repertoire overlap across loci. When comparing the DRB1-restricted epitopes with the ones identified in this study, a strong overlap is shown with DRB3/4/5 loci. In contrast, less overlap was found between DR and DP, and the least minimal overlap of DQ with the other loci. The complete overlap of DRB3/4/5 locus with DRB1 is consistent with the shared used of the alpha chain encoded by the DRA gene. Previous data have shown similarity in binding repertoire between DP and DRB1 loci based on similarities in the peptide-binding motifs (30, 46). In contrast, DQ molecules are associated with more loose peptide-binding motifs and its peptide-binding capability appears more dependent on the main backbone than the specific anchor position giving more importance to the alpha and beta chain combination being both polymorphic and both important in the characterization of the peptide-binding capability (31). Overall the pattern of epitope repertoire overlap is consistent with previous studies by Greenbaum (47) utilizing large collections of peptide binding assays and a collection of purified HLA molecules from different alleles of the DP, DQ, DRB3/4/5, and DRB1 loci. Based on the available knowledge, predictions for the HLA class II molecules in each loci will cover ~50% of the total response, with DRB1-restrictred responses found to be dominant and an appreciable fraction of the responses found instead to be restricted by DP, DQ, and DRB3/4/5 loci (30, 31, 48–50). Our current dataset could not alone evaluate the efficiency of epitope prediction but will provide additional experimental data for future benchmarking of DRB3/4/5, DP, and DQ epitope prediction. Additionally, the present study by defining epitopes restricted by this loci enables future studies to address the relevance of this loci in DENV.

In conclusion, our data highlights that DP, DQ, and DRB3/4/5 restricted are a minor but significant component (as compared to DRB1 restrictions) in DENV-CD4 specific T cell response. This is consistent with lower expression of DP and DQ in APCs, and what has been reported in the general literature for other immunogenic systems highlighting the major role of HLA-DR in triggering CD4+ DENV-specific T cell responses.

## Data Availability

All datasets generated for this study are included in the manuscript and/or the Supplementary Files. All the epitopes generated in this study have been also submitted to IEDB (www.IEDB.org: submission ID:1000789).

## Ethics Statement

Blood samples were obtained from healthy adult blood donors from the National Blood Center, Ministry of Health, Colombo, Sri Lanka in an anonymous fashion. All protocols described in this manuscript were approved by the institutional review boards of both LJI and Medical Faculty, University of Colombo (serving as NIH approved IRB for Genetech).

## Author Contributions

AG, EM, and HV performed experiments, reviewed data, and planned the experimental strategy. JS and BP performed bioinformatics analyses. EP and SM performed and coordinated HLA typing and related analysis. RJ and AMD performed and coordinated serology analysis. ADD collected samples and provided clinical information. AG, DW, and AS conceived and directed the study, and wrote the manuscript. All authors have critically read and edited the manuscript.

### Conflict of Interest Statement

The authors declare that the research was conducted in the absence of any commercial or financial relationships that could be construed as a potential conflict of interest.

## Supplementary Material

Supplementary Table S1 List of the peptides tested in Sri Lanka population for HLA class II studies. The number of peptides is shown per each allele belonging to –DRB3/4/5,-DP, and -DQ, according to DENV protein composition.

Supplementary Table S2 List of the epitopes recognized in Sri Lanka population for HLA class II studies. For each epitope, DRB3/4/5, DP, or DQ allele restriction, DENV deriving protein and response frequency details are shown. All those epitopes have been submitted to IEDB (www.IEDB.org: submission ID: 1000789).

Supplementary Figure S1 Pie chart showing the percentage of the average magnitude per HLA allele calculated in DRB3/4/5 (green), DP (red), DQ (blue), and DRB1 (black) loci.
