# Supplementary figures and images for: Characterization of Magnitude and Antigen Specificity of HLA-DP, DQ, and DRB3/4/5 Restricted DENV-Specific CD4+ T Cell Responses
Source: Front Immunol. 2019 Jul 5;10:1568. doi: 10.3389/fimmu.2019.01568 (PMC6624677; doi:10.3389/fimmu.2019.01568)

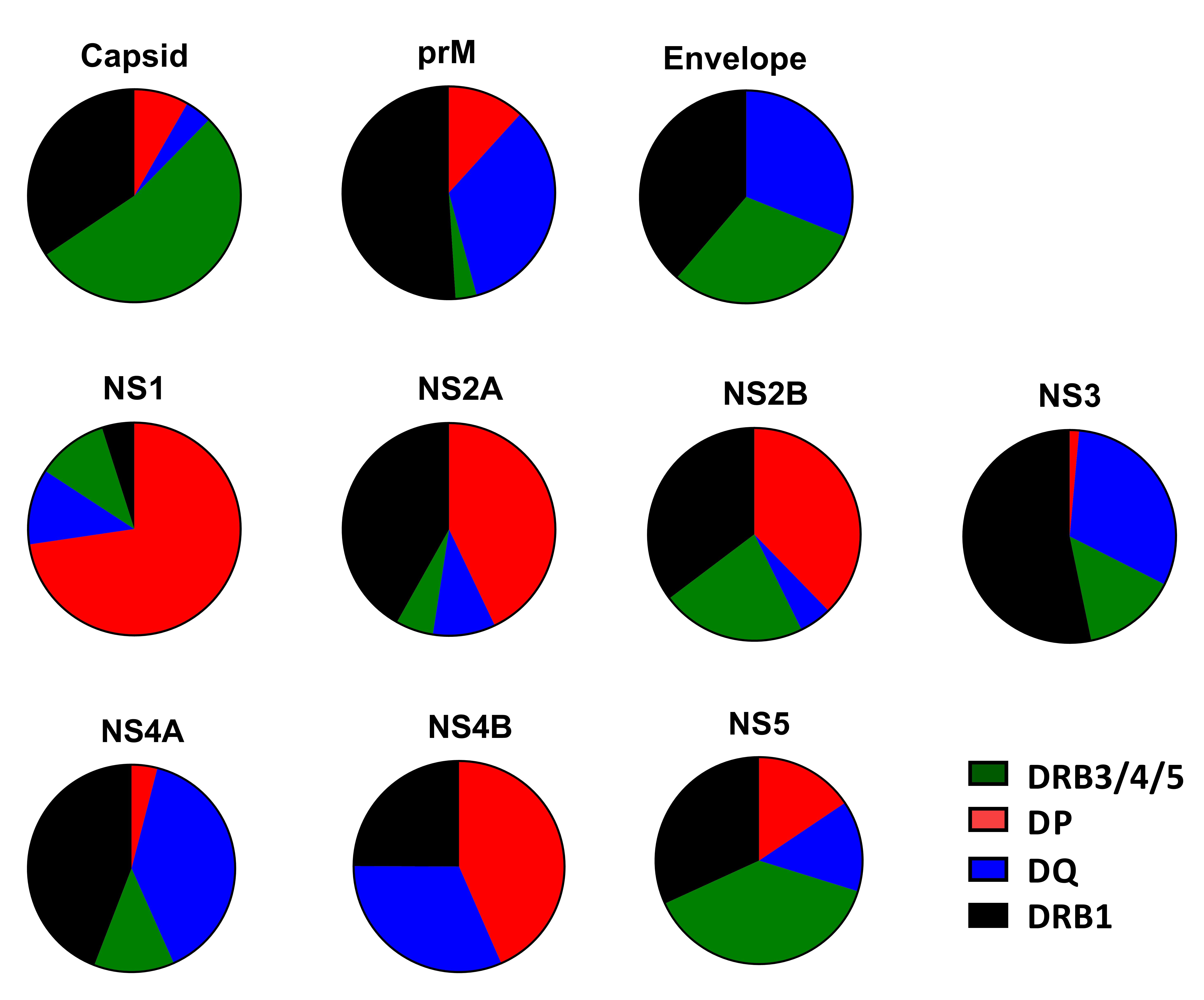

Supplement: Supplementary Figure S1 — Pie chart showing the percentage of the average magnitude per HLA allele calculated in DRB3/4/5 (green), DP (red), DQ (blue), and DRB1 (black) loci. [file Image_1.JPEG]
